# What Is Wrong with the International Holocaust Remembrance Alliance’s Definition of Antisemitism?

**DOI:** 10.1007/s11158-022-09553-4

**Published:** 2022-05-11

**Authors:** Jan Deckers, Jonathan Coulter

**Affiliations:** 1 School of Medical Education, Newcastle University, Newcastle, United Kingdom; 2 Bromley, United Kingdom

**Keywords:** Antisemitism, Ethics, Israel, Palestine, IHRA, JDA

## Abstract

The International Holocaust Remembrance Alliance (IHRA) developed a ‘Working Definition of Antisemitism’ in 2016. Whilst the definition has received a significant amount of media attention, we are not aware of any comprehensive philosophical analysis. This article analyses this definition. We conclude that the definition and its list of examples ought to be rejected. The urgency to do so stems from the fact that pro-Israel activists can and have mobilised the IHRA document for political goals unrelated to tackling antisemitism, notably to stigmatise and silence critics of the Israeli government. This causes widespread self-censorship, has an adverse impact on freedom of speech, and impedes action against the unjust treatment of Palestinians. We also identify intrinsic problems in the way the definition refers to criticism of Israel similar ‘to that leveled against any other country’, ambiguous wording about ‘the power of Jews as a collective’, lack of clarity as to the Jewish people’s ‘right to self-determination’, and its denial of obvious racism. We consider alternative definitions and prefer one like the Oxford English Dictionary (OED) definition, ‘hostility to or prejudice against Jews’, with the addition of the words ‘as Jews’. We recognise that the Jerusalem Declaration on Antisemitism (JDA) can play a useful purpose in illustrating the shortcomings of the IHRA definition. However, we do not advocate promoting it as the prime international definition. Indeed, we question the efficacy of using complex new definitions to combat racism against Jews or other groups, and instead advocate combatting it through collective action across societies.

## Introduction

The International Holocaust Remembrance Alliance (IHRA), a Berlin-based intergovernmental organisation with members from more than 30 countries, adopted a ‘Working Definition of Antisemitism’ at a meeting in May 2016, in Bucharest. The four-line definition is as follows: ‘Antisemitism is a certain perception of Jews, which may be expressed as hatred toward Jews. Rhetorical and physical manifestations of antisemitism are directed toward Jewish or non-Jewish individuals and/or their property, toward Jewish community institutions and religious facilities’ (IHRA 2016).

We find this definition imprecise, starting with the word ‘perception’. It is not clear what is being perceived, and we also wonder whether perceptions can be expressed, as perception involves the act of combining different sensations. It may be more appropriate to say that emotions and beliefs can be expressed. We think that hatred towards Jews can frequently be classed as antisemitic, regardless of what perceptions it might be based on but, by using the word ‘may’, the definition leaves open the question whether antisemitism can also be expressed in different ways.

We believe that the IHRA definition is right to state that ‘antisemitism’ can include ‘hatred toward Jews’, provided that Jews are the object of this hate. This qualification is necessary as ‘hatred toward Jews’ is neither a sufficient nor a necessary condition for antisemitism. It is not a sufficient condition as such hatred may also be motivated by other factors. A misanthrope, for example, hates all human beings, including Jews. An atheist may hate all religious people indiscriminately, including Jews, Christians, and so on. It is not even a necessary condition, as we think that one can be antisemitic without feeling any hate towards Jews. Imagine, for example, someone who is brought up in a community that looks down upon Jews, which might be an unconscious bias. Such a person may not feel any hatred towards Jews, but we think it would be right to call them antisemitic as they would be prejudiced against them, which may or may not lead them to discriminate against Jews.

Robertson (2018) concurs with us here where he reacts against the IHRA definition by stating that ‘by pivoting on expression that arouses hatred (a “very strong word”) it does not cover speech that arouses hostility and fails to protect Jews from many prevalent kinds of antisemitism’. For this reason, Robertson prefers the simple definition of the Oxford English Dictionary (OED): ‘hostility to or prejudice against Jews’. Whilst we believe that this definition is better, we prefer to add the words ‘as Jews’ in light of our argument above.

The IHRA is right to say that antisemitism can be directed against ‘non-Jewish individuals’, for example where some individuals are perceived erroneously to be Jewish, and that one can manifest antisemitism by directing one’s hatred against Jewish ‘property’. However, we think that this additional phrase is confusing and would have best been omitted, given that Jews are the obvious target in such cases.

Readers unengaged with the debate over the definition might be forgiven for thinking that opponents are making a lot of fuss about nothing. However, this definitional lack of clarity poses significant moral issues as it leaves the door ajar both for those who wish to mobilise the definition for unfounded accusations of antisemitism and for instances of antisemitism that are not covered by the definition to go unchallenged. The former problem is illustrated by some of the 11 examples that the IHRA then provides of what it considers ‘may serve as illustrations’ of antisemitism. Significantly, seven of these examples refer to the state of Israel, which suggests that the authors may be largely concerned with anti-Zionism that relates to Israel, as opposed to antisemitism that relates to Jews anywhere in the world.

Whilst it is important to note that the IHRA decision-making body, the Plenary, did not include any of these examples in the definition and that its member countries were only able to reach a consensus on adopting the definition by excluding the examples, the published document that contains the definition nevertheless contains these potential ‘illustrations’. In this regard, Stern-Weiner (2021a, p. 4) finds that the ‘senior IHRA officials and pro-Israel groups’ that were involved in the publication of this document ‘have misrepresented the IHRA Plenary’s decision in order to smuggle into the Working Definition examples that can be used to protect Israel from criticism’ and pressurised governments and other organisations to adopt it.

Such pressure has been particularly evident in the case of the UK government (2016), which formally adopted the definition in 2016. In October 2020, Gavin Williamson (2020), Secretary of State for Education in England, urged higher education institutions to follow suit in adopting the definition with its examples, writing that the ‘definition helps us better understand and recognise instances of antisemitism’. Most significantly, he threatened to suspend funding streams for universities that did not sign up to the definition (Adams 2020; Harpin 2020). The threat appears to have borne fruit, since Hazel (2021) reports that by November 2021, and after some initial hesitation, 216 higher education providers had signed up. This includes 95 out of a total of 133 universities which, on the face of it, is a remarkable increase on the 28 universities that had signed up by September 2020. However, according to the Palestine Solidarity Campaign and the British Committee for Universities of Palestine (PSC and BRICUP 2021), ‘a high proportion of those universities that have in some sense said “yes” to the IHRA definition have done so using forms of words that keep some distance–not “adopt”, but “acknowledge”, or “recognise”. Many have asserted the adequacy of their pre-existing policies on hate speech. Some have added caveats to their adoption to seek, however inadequately, to protect academic freedom from the definition’.

Whilst many universities have fallen in line with Williamson’s demand, several legal scholars have questioned the rationale. Sedley (2017), for example, has claimed that ‘the official adoption of the definition, while not a source of law, gives respectability and encouragement to forms of intolerance which are themselves contrary to law, and higher education institutions in particular need to be aware of this’. In a similar vein, Gould (2018, p. 24) writes that ‘by singling out a specific group for protection that is not accorded to others … the law’s commitment to equality for all’ is called into question. Shlaim (2021) describes Williamson’s pressure on English universities as a threat to free speech, which universities and the Department for Education have a statutory duty to uphold. Muhareb et al. (2021) provide a recent example showing how this threat came to fruition when the *Lancet* published, and then withdrew, a letter these authors had written, alerting the medical community to the dangers of a COVID-19 outbreak in the Gaza Strip.

In the next two sections, we comment on the examples that accompany the definition. We follow up by examining the impact that the IHRA definition is having in the UK and other Western countries, after which we discuss alternative definitions that might be used.

## Are the Potential Illustrations of Antisemitism Antisemitic?

The IHRA definition recognises rightly that some examples that could be antisemitic may not necessarily be antisemitic. We illustrate this below by discussing three examples.

In its fourth example, the IHRA mentions Holocaust denialism, which is likely to be motivated by hatred towards Jews (Kuper 2020). However, it might possibly be motivated by people living with delusions, for example a German denying that World War II took place because of a psychological inability to accept as facts the crimes perpetrated by their parents, or an individual’s proclivity for conspiracy theories of all kinds, not just those that concern Jews. He or she may believe that many things that are widely accepted as historical facts are, in fact, created by those who are ‘engaged in a complex and secretive conspiracy’ (Diethelm and McKee 2009).

In its sixth example, the IHRA also mentions ‘accusing Jewish citizens of being more loyal to Israel … than to the interests of their own nations’. The IHRA is right that this is not necessarily antisemitic. It would be antisemitic to say that all Jews, or Jews in general, are more loyal to Israel. However, those who argue that some Jews are more loyal to Israel, and who take issue with this, are not necessarily wrong. The persistent and determined lobbying activity of many Zionist Jews in Western nations might stem from their greater loyalty to the state of Israel. By the same token, it could be argued that there are UK and USA citizens of Irish origin or descent who have been more loyal to the Republic of Ireland than to the UK or the USA and who have shown this by their willingness to finance or organise violence in Great Britain and Northern Ireland. In today’s world, with millions of people living far from their ancestral homes, many citizens feel conflicting loyalties.

The tenth example is ‘drawing comparisons of contemporary Israeli policy to that of the Nazis’. We agree that this is not necessarily antisemitic. Broad unqualified statements treating Nazism and Zionism as similar, or equally bad, are likely to spring from antisemitism and/or gross ignorance. However, historians and others who study these and other ethno-nationalist movements of European origin, for example Serbian nationalism, may quite reasonably identify certain historical parallels along with major dissimilarities.

Despite the IHRA’s recognition that some examples are not necessarily indicative of antisemitism, those advocating for Israel typically refer to the list of examples as unequivocal instances of antisemitism, using a simplistic matching process: they match the behaviour of the accused to one of the examples, without considering context and whether the motivation is truly antisemitic. This can be seen very clearly on the web pages of the Campaign Against Antisemitism (CAA 2022), which has assumed the mantle of monitoring and enforcing the implementation of the IHRA definition in the UK. It particularly focuses on ensuring compliance by the political parties, the universities, and local authorities. In the case of the political parties, CAA has a section for each political party, and dedicates pages to individual members and sometimes ex-members, where it lists what it calls antisemitic ‘incidents’. It mainly focuses on the Labour party, for which it listed, as of 17 January 2022, 40 members and 170 ‘incidents’. It also has pages for other parties, including nine for the Greens, seven for Plaid Cymru, six for Conservatives, four for Liberal Democrats, three for the Scottish National Party (SNP), three for the Brexit Party and one for the UK Independence Party (UKIP). We reviewed the CAA’s pages for the 19 Labour members, including Jeremy Corbyn, Diane Abbott, Chris Williamson, and the late Gerald Kaufman, to whom CAA attributes three or more incidents, finding that without exception it uses the simplistic matching process mentioned above, to declare the individual to have breached what they call ‘the International Definition’ (i.e. the IHRA definition), without discussion of context or whether the motivation was truly antisemitic.

The unequivocal interpretation of the examples is also evident in a message that Luke Akehurst MP, the Director of an organisation called ‘We Believe in Israel’ and a member of the National Executive Committee of the Labour Party, sent to British local authorities. Cushman (2017) shows that Akehurst circulated an edited version of the IHRA definition without telling his recipients that it had been edited. The introductory words of the IHRA document stated that the examples ‘could, taking account of the overall context, include …’, but Akehurst’s version simply read: ‘The guidelines highlight manifestations of antisemitism as including: …’.

Many observers attribute such sleights of hand to pro-Israel advocates seeking to clamp down on people who criticise the conduct of the Israeli government (see for example: Winstanley 2020; Stern-Weiner and Maddison 2019; Stern-Weiner 2021b). One of the original drafters of the IHRA definition, Kenneth Stern, accused right-wing Jews of weaponising it. According to Stern’s testimony, these advocates have been enormously persistent in their quest to close down free speech on Israel in the USA: ‘The Zionist Organisation of America (ZOA) and other groups will hunt political speech with which they disagree and threaten to bring legal cases’ (Stern 2019). Further on in this article, we show in greater depth how the IHRA definition has been instrumentalised to shield the Israeli government from criticism as well as to falsely frame pro-Palestinian activists as antisemitic.

## Criticism of the Israeli Government

Whilst we have argued in the previous section that some aspects of the IHRA document are relatively trouble-free due to the recognition of the importance of context, other aspects of the document are problematic per se. Whilst introducing the 11 examples, the IHRA definition states that ‘criticism of Israel similar to that leveled against any other country cannot be regarded as antisemitic’. This statement fails to explain why greater criticism of one state compared to another would be problematic, and questionably implies that dissimilar criticism of Israel can ‘be regarded as antisemitic’.

To evaluate this claim, it must be clarified what is meant by criticism of a country. Does criticising a country mean criticising all those who live within its borders, those who are recognised as its nationals, or those who govern it? Any criticism of the first two categories is likely to involve criticising people who bear no responsibility for the act that is being criticised. The same applies to the third option, with the difference that, when one criticises a government, a well-defined body is being criticised, where it is understood that not all who are part of the government necessarily support its decisions.

If ‘criticism of Israel’ is understood in this way, it is not clear why criticism dissimilar ‘to that leveled against any other country’ can ‘be regarded as antisemitic’. The concept of dissimilar criticism is problematically vague as it can mean that the same standard of criticism is applied to different situations or that a different standard is applied to the same kind of situation. It seems particularly inappropriate to associate the former with antisemitism. Firstly, the criticism might not be motivated by hostility or bias against Jews, but simply by a particular government policy. Secondly, if government A has more unjust policies compared to government B, there is good reason to subject it to more criticism. There is good reason, for example, to criticise the Israeli government more than any other government in relation to the plight of Palestinians. A recent report by Human Rights Watch (2021, p. 353) states that ‘Israeli authorities in 2020 systematically repressed and discriminated against Palestinians’, for example through ‘the further transfer of Israeli citizens into settlements in the occupied West Bank’ and the demolition of Palestinian homes ‘for lacking Israeli building permits, which are virtually impossible to obtain’.

It might be objected that it would not be fair to criticise the Israeli government for this unless one makes similar efforts to criticise other governments that also engage in human rights violations, for example China in relation to the Uighurs, Myanmar in relation to Rohingyas, and Syria in relation to the death and suffering of its own people. Those who thus object are treating any criticism that is dissimilar as morally problematic and, in the case of the Israeli government, suspect of being motivated by antisemitism, as one should only speak out against a particular injustice if one speaks out simultaneously against all injustices. As no person is capable of expressing criticism against all injustices equally, this would lead to the absurd situation where all criticism would be invalid. Moreover, we are at one with those who argue that people are entitled to focus more attention on human rights problems where their own governments have a particular historical responsibility, particularly where this resulted in enduring injustice (Spinner-Halev 2007), and where one has considerable leverage to alleviate the injustice (Caney 2014). In this regard, we note that the United Kingdom has considerable historical responsibility for the Israeli-Palestinian dispute, which goes back to the Balfour Declaration of 1917 and the League of Nations Mandate, and that it has much more leverage over Israel than over territories such as Xinjiang, that lies within the Peoples’ Republic of China, or Myanmar that shares a border with it. The Syrian conflict has caused untold misery, but Britons may say that their government has limited leverage, given the number of state and non-state actors involved, including Russia, a nuclear-armed nation. We therefore reject this objection.

## Ambiguous Wording about the Exercise of Power by Jews

In its second example, the IHRA document also describes as potentially antisemitic: ‘Making mendacious, dehumanizing, demonizing, or stereotypical allegations about Jews as such or the power of Jews as collective—such as, especially but not exclusively, the myth about a world Jewish conspiracy or of Jews controlling the media, economy, government or other societal institutions’. The problem with this example lies in the ambiguity of the word ‘collective’ in relation to the word ‘myth’ that follows shortly afterwards.

According to one interpretation, the example could be describing the idea that all Jews worldwide are involved in a ‘Jewish conspiracy’ or are ‘controlling the media, economy, government and other societal institutions’. The idea that all Jews or Jews as a whole control the institutional life of nations of which they constitute small minorities is certainly a racist stereotype or an overgeneralised belief, and one that was repeatedly used maliciously during the first half of the 20th century, notably with the fraudulent ‘protocols of the Elders of Zion’, first published in Tsarist Russia in 1903 (Hagemeister 2008). This is clearly a myth.

However, the idea that some collectives of Jews, for example the organisations composing the pro-Israel lobbies of Western countries, exercise strong influence over some media and other institutions is clearly not a myth. The question of how much influence these or any other interest groups exercise within our societal institutions is an empirical matter, and should not be decided by fiat. It is undeniably the case that farmers, gun-owners, media magnates, and petrochemical companies exert significant influence over political institutions, and it would be absurd to assert that Jewish lobby groups are fundamentally different from other humans in this regard.

One only needs to acknowledge the power of the pro-Israel lobby organisations such as the America Israel Public Affairs Committee (AIPAC) and the Anti-Defamation League in the USA, the Jewish Board of Deputies and Conservative Friends of Israel (CFI) in the UK, and Conseil Représentatif des Institutions Juives de France (CRIF), to name a few. Such groups do not represent the views of all Jews, many of whom are opposed to Zionism, as have been many Jews since the inception of the Zionist movement at the end of the 19th century. However, between them, these organisations exercise significant power over some areas of institutional life in their respective countries. In the USA, for example, Mearsheimer and Walt (2007) have shown that the pro-Israeli lobby, a ‘loose coalition of individuals and organizations’, has steered USA foreign policy in a pro-Israel direction. Weir (2014, pp. 85–90) drew on a variety of primary sources to show how Zionist groups managed to gain influence in the American media in the 1940s. Given Benjamin Netanyahu’s boast about Israel’s power over the United States (Huffington Post 2011; Kessler 2010), the idea that Zionist groups exert considerable power within the USA is clearly not a myth.

In the UK, for example, CFI is a particularly influential organisation, claiming 2,000 members, including 80% of Conservative MPs (see CFI 2014; Teller 2020). In 2016, it used its influence to block the appointment of Alan Duncan, a leading critic of Israel, to Foreign Office responsibilities for Middle Eastern matters excluding the Sultanate of Oman (Duncan 2021). Another influential group in the UK is the CAA, which has persistently and unerringly opted for the second interpretation, as we found in its pages listing the antisemitic ‘incidents’ of British politicians. All 19 pages about the Labour party members that we reviewed cited the second example as cause for declaring them to have breached ‘the International Definition’, more than twice the number of any other example cited. CAA supported the long-running campaign against David Miller, a professor of sociology, that eventually led to his dismissal from the University of Bristol. Miller has been outspoken about the power exercised by the Zionist movement within the UK and elsewhere, but we have seen no evidence that he has criticised Jews simply because they are Jews. However, for CAA (2019), this is one more case of an antisemite denouncing the power of Jews as a collective, and it sums this up by stating that ‘the idea that Jews secretly exert extraordinary power over society and hold dual loyalties are longstanding antisemitic canards’.

An example of how pro-Israel advocates seek to discredit those who would discuss the power that they themselves exercise can be found in the House of Lords Commissioner for Standards report on ‘The Conduct of Baroness Tonge’, as Chair of a Public Meeting to Launch a ‘Balfour Apology Campaign’ on 25 October 2016. The Commissioner received complaints from six members of the House about a speaker who said that a Zionist lobby had power over Parliament, which they described as ‘a modernised version of a classic antisemitic trope’ (House of Lords 2017, Annex 2, paras 6, 40–43). In this case, the Commissioner rejected the view that the claim that a particular Zionist lobby exercises significant power should be interpreted as antisemitic.

## Self-determination and ‘Racist Endeavours’

The seventh example, ‘denying the Jewish people their right to self-determination, e.g., by claiming that the existence of a State of Israel is a racist endeavor’, is also highly questionable. We treat the Jewish people here as an ethnic group of which some practise Judaism, whilst others are secular, agnostic, or atheistic. It is an open question that we do not wish to discuss here whether and how far Jews can be considered a single ethnicity or a heterogeneous group of people which has, in the past, been united in their religious beliefs and practices. For the purpose of this discussion, we treat them as a single ethnicity.

The right to self-determination is embodied in article 1 of the United Nations Charter (1945), which states (par. 2) that the UN aims ‘to develop friendly relations among nations based on respect for the principle of equal rights and self-determination of peoples’. More detail is provided in the International Covenant on Civil and Political Rights, which states: ‘All peoples have the right of self-determination. By virtue of that right they freely determine their political status and freely pursue their economic, social and cultural development’ (United Nations 1966, article 1, par. 1). This UN document does not specify that the right of self-determination applies in relation to the possession of a particular territory. However, we think that it is reasonable, in terms of international law, to interpret it as applying to the exercise of control over some territory (Ojukwu and Okoli 2021). Assuming that this is a valid interpretation, it is still unclear over which territory ‘the Jewish people’ might be claimed to have a right to self-determination. As pointed out by Goldberg (2019, p. 244), it could be ‘the territory recognised by the international community, the territory claimed by the State of Israel, or the Land of Israel from Wadi of Egypt to the great river, the Euphrates (Genesis 15:18)’.

Crucially, one is not necessarily antisemitic ‘by claiming that the existence of a State of Israel is a racist endeavor’. On a charitable reading of this claim, one might read this as saying that the existence of any state of Israel would be racist, where we understand ‘racism’ as a prejudice that motivates discrimination based on one’s belonging to a particular racial or ethnic group. If so, we think that a charge of antisemitism might be appropriate, provided that Jews are the citizens of such a state and the targets of such a comment.

On the other hand, the claim might be read in terms of the view that the actual state of Israel is racist. To assess the veracity of such a claim, one must assess whether the social and political structures of that state, such as its Constitution, policies and institutions, are infused with racist ideas. The widespread, institutionalised racial discrimination in Israel, of which the Nation State Law (Government of Israel 2018) is merely one prominent example, suggests that the state of Israel is racist. Principle 1(c) of the Nation State Law states: ‘The exercise of the right to national self-determination in the State of Israel is unique to the Jewish People’. This excludes those who belong to other ethnic groups which have been living there for centuries from the right to national self-determination. Adalah (2022) cites 70 other discriminatory laws, while ex-Prime Minister Netanyahu has asserted that ‘Israel is not a state of all its citizens … Israel is the nation-state of the Jewish people and them alone’ (Allen 2021). Given this and authoritative testimonies about the existence of apartheid (Allen 2021), it is difficult to resist the conclusion that the actual Israeli state is indeed racist.

Notwithstanding, some pro-Israel advocates use this example to discipline critics within the UK and elsewhere. In our review of the CAA pages on 19 Labour party members, we found that it declared seven of them in breach of the IHRA definition for ‘denying Jewish people their right to self-determination’. Shlaim (2021) cites the case where the Labour party ruled a local party branch out of order when it tried to submit a motion endorsing B’Tselem’s latest report alleging the existence of apartheid in Israel because, according to the IHRA definition, this could be seen as designating Israel a ‘racist endeavor’. In these cases, it is clear that those issuing these strictures are referring to the actual state of Israel, not to any state that might exist hypothetically.

## How the IHRA Definition Is Being Used in Western Countries

In this section we show in greater depth how the IHRA definition has been instrumentalised to shield the Israeli government from criticism and to falsely frame pro-Palestinian activists as antisemitic. We start by examining three multi-country reviews, after which we look more closely at the situation in the UK and North America.

Goldberg et al. (2021, pp. 12–16) provide a long list of examples, while citing respective references. The targets of the instrumentalisation include the European Union, European governments and parliamentarians, the UN and the International Criminal Court (ICC), human rights and humanitarian organisations, the Boycott, Divestment and Sanctions (BDS) movement, anti-Zionist activists, academics critical of Israel, online activity critical of Israel, Facebook, and NGOs that criticise and challenge the Israeli government. The definition was also being cited as contributing to the ‘deplatforming of activists and events, chilling free speech, constraining academic freedom’ (Goldberg et al. 2021, p. 16).

Goldberg et al. (2021, pp. 14–16) show that pro-Israel advocates have been portraying BDS and anti-Zionism as categorically antisemitic, while seeking to enshrine the IHRA into law and to incorporate the IHRA definition into the training of attorneys and judges, into university codes of conduct, and antisemitism guidelines. In this regard, they cite Gould (2017, p. 1), who examined how the IHRA definition has been applied within the UK following its adoption by the British government, saying that the document had reached beyond its self-described status as a ‘non-legally binding working definition’ and had come to function as a quasi-law, in which capacity it exercises the *de facto* authority of the law, without having acquired legal legitimacy.

European Jews for a Just Peace (2021, p. 1), representing 12 Jewish groups in seven EU member states, the UK, and Switzerland, also submitted evidence to the European Commission, writing that ‘since 2016, the big, communal Jewish organisations, supported by the Israeli government, have been actively promoting IHRA and its associated examples. The WD (Working Definition) and the examples are worded in such a way as to create confusion between criticism of Israel and antisemitism, and are used by its proponents to claim that much fundamental criticism of Israel is antisemitic’. The group was also critical of the European Commission for relying on the IHRA definition and examples to judge what is or is not antisemitic, for promoting a Handbook for the Practical use of the IHRA (European Commission 2021), and encouraging its use for funding-related purposes. It goes on to assert that this runs counter to the Commission’s strategy for the protection of freedom of expression.

Independent Jewish Voices Canada (2021) documented examples in several countries of where the IHRA definition and examples have been cited, or significant attempts have been made, to cancel events or silence Palestine solidarity movements. Its latest report shows cases in eight Western countries up to 4 November 2021, and particularly focuses on events at 15 universities in the United States.

In the UK, we have drawn attention to the CAA which, along with Community Security Trust (CST) and a host of other pro-Israel organisations and bloggers, strongly support the IHRA definition. As indicated above, we reviewed the CAA web pages for 19 Labour members that the CAA charged with three or more ‘incidents’ of antisemitism. In the case of 17 of these members, CAA used their tweets and Facebook posts to certify ‘incidents’. Indeed, CAA very frequently holds members accountable for sharing, ‘liking’, or supporting other individuals whom CAA had judged to be antisemitic, something one might describe as ‘guilt by association’.

As regards the motives underlying members’ ‘incidents’, all but one (18) expressed serious concern about pro-Israeli advocates defaming Labour members on grounds of antisemitism, in what they saw as a systematic campaign to discredit the Labour party under the leadership of Jeremy Corbyn. At least 11 expressed their opposition to Zionist ideology and/or the situation in Israel and Palestine. Four members, including Matthew Collings, Bill Curran, Pamela Fitzpatrick, and Kate Hollern were critical of the scant attention this campaign had paid to racial prejudice on the right and within the Conservative party.

These people were not making indiscriminate generalisations about all Jews, and their testimonies show considerable awareness of the many non-Zionist Jews who oppose the IHRA definition. Some of their language might be considered provocative, and occasionally uncouth; this was particularly the case of one young member who made some offensive tweets while still in his teens, and about which he has since apologised. Overwhelmingly, however, members’ statements spring from their objection to the heavy and sustained publicity about alleged antisemitism in Labour in the mainstream media ever since their leader, Jeremy Corbyn, became leader of the Labour party in 2015, coupled with statistical and other evidence that belies the mainstream narrative (Stern-Weiner and Maddison 2019; Jewish Voice for Labour 2019; Kennard and Curtis 2019; Philo et al. 2019). The CAA therefore has no basis for asserting that it stems from antipathy towards Jews as an ethnicity.

The CAA accused all 19 people of being in breach of the IHRA definition, all of them based on example 2 (‘making mendacious, dehumanizing, demonizing, or stereotypical allegations about Jews as such or the power of Jews as collective, …’), nine based on example 10 (‘drawing comparisons of contemporary Israeli policy to that of the Nazis’), seven based on example 7 (‘denying the Jewish people their right to self-determination’), three on example 6 (‘accusing Jews of being more loyal to Israel, …’) and one on example 3. We would argue that CAA’s reasoning is a form of ‘double-think’; by pivoting its accusations first and foremost on example 2, which is about Jews exercising power over societal institutions, it is in fact objecting to people speaking of the power that CAA and similar pro-Israeli organisations themselves (seek to) exercise. CAA objects to Matthew Collings’s statement about the existence of an ‘Israel Lobby’, calling it a ‘trope’ about the hidden power of diaspora Jews or Israel, and makes related comments about Jeremy Corbyn, Bill Curran, Rebecca Gordon-Nesbit, Kate Linnegar, and Lloyd Russell-Moyle. These comments by CAA show it to be in denial about its own role as a lobbyist.

Those lobbying for Israel, including the CAA, make many complaints about what they consider antisemitic behaviour, and the constant pressure is not without consequences. Several of the Labour people accused faced deselection penalties in the form of suspension, expulsion, and being pressurised to apologise or to resign from their party posts. Along with the Jewish Labour Movement, CAA made the complaints that prompted the Equality and Human Rights Commission (2020, p. 16) ‘Investigation into Antisemitism in the Labour Party’ that reported in October 2020. However, it sometimes encounters difficulty accusing people with a strong legal background, as when it took on the barrister Franck Magennis (CAA 2021).

In 2019, council officers for the London Borough of Tower Hamlets cited the IHRA definition when refusing permission for the ‘Big Ride for Palestine’ to stage an event in the Borough. The Big Ride is a cycling organisation that organises sponsored rides that raise funds for a Palestinian children’s charity. Members of two local constituency Labour parties then proposed motions in support of the Big Ride, but these were ruled out of order, reportedly on instructions from the General Secretary of the Labour party, which has adopted the IHRA definition and all its examples (Skwawkbox 2020).

A group of researchers sponsored by the Balfour Project conducted some interesting research that tells us how the IHRA definition is being perceived by potentially interested parties on UK campuses where it has been rolled out (Lukman et al. 2021). Their study methodology involved in-depth online interviews with 33 anonymous respondents, ‘including Jewish students and academics, members of Palestinian Solidarity groups and community activists’ (Lukman et al. 2021, p. 4). Some respondents expressed considerable reservations about the IHRA definition, mainly because it fosters a climate of ‘implicit coercion’ to steer clear from some themes related to Israel/Palestine (Lukman et al. 2021, p. 5), thus recognising that it is causing self-censorship and fear (see also: Himmo 2021). Other themes that respondents identified include how organisational pressure to use the definition is perceived as jeopardising professional careers of researchers who study Palestine and discouraging some from getting involved in pro-Palestinian activism. The authors support these themes with many examples from the respondents’ personal experiences.

One of the most interesting findings is that understandings of antisemitism differ greatly among Jews at UK universities, with many expressing reservations or discomfort about the idea of a universal or objective definition of antisemitism. Some respondents also feared that the insistance on adopting the definition might unjustifiably prioritise confronting antisemitism more than other forms of discrimination, such as Islamophobia. Many respondents believed that a broader programme of education and cross-community work is needed to tackle antisemitism in higher education (Lukman et al. 2021).

Other authors cite cases where the IHRA definition has been instrumentalised at UK universities. Gayle (2017) cites the case of Michael Freeman, the Counsellor for Civil Society Affairs at the Embassy of Israel in the UK, who pressurised the University of Manchester into changing the title of a talk that was critical of the government of Israel, whilst invoking the IHRA definition. Gould (2018) documents several examples where British universities invoked the IHRA definition to modify the content of, or close down, events connected with ‘Israel Apartheid Week’. Shlaim (2021) mentions a campaign of character assassination against Ken Loach, the high-profile film director who had long stood up for the Palestinians, including demands that his former Oxford college cancel an event at which he was to speak.

In the USA, Palestine Legal is a major source of information on the instrumentalisation of the IHRA definition. Palestine Legal (2020a) says it is routinely used to chill campus speech and provides examples from six universities. Palestine Legal (2020b) shows that Israel advocates had escalated a campaign to push local, state, and national governments, as well as universities, and more recently social media companies, to adopt the IHRA definition. The social media initiative is described by Friedman (2020). In a later article, Palestine Legal (2021) tells us of multiple attempts to get Palestine advocates fired from their jobs or expelled from universities. The USA has robust constitutional protections, based on the First Amendment, guaranteeing free speech, but according to Palestine Legal (2020a), this does not deter numerous attempts to get the IHRA definition incorporated into USA law and policy. Neither, according to Palestine Legal (2020b), did it stop the US Special Envoy to Monitor and Combat Antisemitism, Elan Carr, from invoking the IHRA definition when criticising ‘certain non-Governmental organisations’ for supporting the BDS movement.

In Canada there is considerable concern that the IHRA document is being used to shield Israel from criticism. Sachs (2019) provides a succinct summary of how the IHRA definition has been mobilised to silence criticism of Israel in both Canada and the United States, particularly on university campuses, and warned of the likelihood of similar episodes in the future. Among other cases, Areguy (2021) cites the reaction of pro-Israel groups to a politician who had retweeted an article criticising Israel’s decision not to distribute COVID-19 vaccines to Palestinians living under its occupation. Gadzo (2020) reports a controversy around the University of Toronto’s Faculty of Law rescinding a job offer to Valentina Azarova, who was to serve as Director of its international human rights programme, at a time when pro-Israel groups were intensifying their pressure to get universities to adopt the IHRA definition. Some faculty accused the Dean of rescinding a job offer because a major donor was uncomfortable with her scholarly criticism of Israel’s human rights record. The decision led to a series of resignations at the University.

The mobilisation of the IHRA document to curtail free speech by critics needs to be seen in the context of the government of Israel’s close relationships with right-wing politicians in Western countries, as well as right-wing regimes that turn a blind eye to antisemitism but support Israel, such as those in Hungary and Poland (Shroufi 2015; Lerman 2018; Sternhell 2019). These authors show that the underlying motive for this alignment is the existence of common enemies. For example, Sternhell (2019) asserts that for some Poles and Hungarians, ‘Israel has become a state with which white racists in Europe can identify’, and that ‘far-right Europeans feel they can learn from Israel how to deal with strangers from Africa and local Muslims’. He goes on to say that President Netanyahu ‘has supported Hungary’s populist prime minister, Viktor Orbán, who has mobilised dangerous antisemitic sentiment against the liberal Jewish philanthropist George Soros’, and ‘has given the Polish government a free pass on its legislation criminalising opinion and scholarship which shows that Polish people were complicit in the Holocaust’. In view of this alignment between the government of Israel and the right-wing of Western countries, one may reasonably ask whether the mobilisation of the IHRA document is really motivated by a desire to combat antisemitism. Both Shlaim (2021) and Stern-Weiner (2021b) think it is more concerned with the protection of Israel. We concur.

## Alternative Definitions of Antisemitism

As explained above, Robertson (2018) prefers the OED definition, i.e. ‘hostility to or prejudice against Jews’, to which we would propose adding the words ‘as Jews’. An alternative to this is the Jerusalem Declaration on Antisemitism (2021). An international group of scholars created this two-page document with the aim of providing clear guidance to those seeking to identify and fight antisemitism, while at the same protecting free expression. The authors describe it as an international definition, or in their own words ‘a tool to identify, confront and raise awareness about antisemitism as it manifests in countries around the world today’. It starts with a preamble, followed by a very concise basic definition: ‘discrimination, prejudice, hostility or violence against Jews as Jews (or Jewish institutions as Jewish)’. This is followed by 15 guidelines, five of which are ‘general’, five about Israel and Palestine and ‘on the face of it are antisemitic’, and five about Israel and Palestine and ‘on the face of it, are not antisemitic’.

The JDA is a massive improvement on the IHRA definition. It avoids referring to the concept of perception, broadens the scope from hatred, adds the central qualifier ‘as Jews’, recognises antisemitism as a form of racism, and rejects the idea that criticising Israel or boycotting the country is antisemitic. Several of the guidelines can be read one-to-one as ‘bug fixes’ to the IHRA examples. The authors of the JDA explicitly rejected codifying it into law, using it to restrict the legitimate exercise of academic freedom or to ‘suppress free and open public debate that is within the limits laid down by laws governing hate crime’.

Notwithstanding, various authors have taken issue with the JDA, notably the Palestinian BDS National Committee (2021), Englert (2021), Shihadah (2021), and Suárez (2021). Their leading criticism is that this avowedly international definition discusses antisemitism overwhelmingly in the context of the Israeli-Palestinian struggle, but never mentions the far right which has historically been the main home of antisemitism around the world, and which in the present day is overwhelmingly supportive of Israel and its regime of oppression (see comment at the end of the last section on Israel’s close relationship with right-wing politicians and regimes). Englert (2021) describes the far right as the main risk to Jews, notably in Europe and North America, where there has been a rise of xenophobic parties, whilst Suárez (2021) makes this comment: ‘true anti-Jewish bigots, the presumed subject of the definition, are far more likely to be pro-Israel and wholly supportive of its “human rights violations”’. We believe that these authors rightly question whether such a focus on Israel and Palestine is warranted in an international definition.

The Palestinian BDS National Committee (2021) claims that the JDA excludes Palestinian perspectives on a conflict characterised by asymmetric relations of power. It also expresses the fear that some of the JDA’s ‘guidelines’ will be used ‘to police speech critical of Israel’s policies and practices, failing to fully uphold the necessary distinction between hostility to or prejudice against Jews on the one hand and legitimate opposition to Israeli policies, ideology and’ its ‘system of injustice on the other’.

Given the virtues of the JDA noted above, the fear expressed by the BDS National Committee and other critics may not be altogether reasonable, but there is no question that they reveal deep-seated mistrust, based on over 70 years’ worth of legitimate grievances–deriving from the actions of the state of Israel and its driving Zionist ideology. In this light, we think that the JDA may have certain uses, notably as a tool for illustrating the shortcomings of the IHRA definition, but that it would be unwise to promote it as the prime international definition. It risks evoking widespread mistrust and opposition among Palestinians and other people against whom many supporters of Israel have unjustly wielded the IHRA definition.

We question the efficacy of creating radically new definitions to combat racism against Jews or other groups. Inasmuch as a definition is needed, we recommend using one like the OED definition, as it has advantages in terms of simplicity and equality. As regards simplicity, it requires those asserting the existence of antisemitism to go back to first principles. By citing evidence and simple reasoning, they can identify: (a) irrational and untruthful generalisations about the attitudes and behaviours of Jews in general (e.g. ‘they are only interested in money’, ‘they control the world’, or ‘they only care about Israel and not about the countries where they live’); (b) people calling for or participating in the harming of Jews; (c) Holocaust denial, and; (d) depending on context, using symbols and images associated with classical antisemitism to characterise Jews. All these are likely to spring from anti-Jewish animus and relate directly to the OED’s words ‘hostility to or prejudice against Jews’.

As regards equality, the OED is simply a definition of prejudice or discrimination against Jews, one that can equally be applied to other groups (e.g. Christians, Muslims, Romani people, Palestinians, LGBT + people, etc.) simply by substituting for the word ‘Jews’. By contrast, bespoke definitions containing examples, like the IHRA definition, risk creating a hierarchy of racism and discrimination against minorities and fragment the worldwide fight against these phenomena. In this regard, Englert (2021) states rightly: ‘In aiming to address racism through definitions, this approach sidesteps the necessary work of developing collective action across societies’.

## Conclusion

The IHRA’s four-line definition of antisemitism correctly states that hate towards Jews may be antisemitic but is worded vaguely and inadequately, in a way that opens the door to unfounded accusations of antisemitism and allows other instances to go unchallenged. However, the main problems lie with the examples that it provides of what it considers ‘may serve as illustrations’ of antisemitism. Seven of the 11 examples explicitly refer to the state of Israel.

The IHRA acknowledges that behaviour that fits some of the examples that it lists may not necessarily be antisemitic. However, in practice, many pro-Israeli advocates use them as unequivocal examples of antisemitism, with a view to clamping down on those who criticise the conduct of the government of Israel. Some aspects of the definition are particularly problematic. It is not clear why criticism of Israel dissimilar ‘to that leveled against any other country’ can ‘be regarded as antisemitic.’ Ambiguous wording about ‘the power of Jews as a collective’ encourages pro-Israel lobbyists to attack and discredit those who would discuss the power that they themselves exercise. The ‘right to self-determination of the Jewish people’ is clouded by a lack of clarity as to the territory over which Israel is claimed to have a right of self-determination. The assertion that ‘the existence of a State of Israel is a racist endeavor’ is not necessarily antisemitic; indeed, given its current constitutional, policy, and institutional framework, and the way this is applied in practice, the actual state of Israel is racist.

Rather than being an instrument to promote tolerance, we have argued that the IHRA document can be misused for political goals unrelated to tackling antisemitism, notably to stigmatise and silence critics of the Israeli government’s treatment of indigenous Palestinians, and we have shown that many pro-Israel advocates have been doing just this. The uncritical acceptance of this document not only impacts on the lives of Palestinians, but on the right to freedom of expression elsewhere. It risks placing the onus on the accused to prove that he or she is not antisemitic and creating a minefield for people who wish to speak about Israel, encouraging self-censorship and the cancellation of public events that might be critical of Israel.

The definition has already been widely adopted in educational institutions in the UK and elsewhere and is seriously threatening academic freedom on topics concerning Israel. In view of this, we recommend that institutions resist pressure to adopt, or revoke, the definition and the examples associated with it, following the well-documented example of the University College Academic Board (2020) of the University of London.

We question the efficacy of creating definitions as a means of combating racism against Jews or other groups and believe this approach sidesteps the necessary work of developing collective action across societies. Inasmuch as an international definition is needed, we recommend using one like the OED, which is advantageous in terms of its simplicity and in helping to achieve equality among groups subjected to racism and discrimination. We think that the JDA can prove useful as an educational device, particularly in highlighting flaws of the IHRA definition, but we have argued that there are good reasons not to recommend it as the prime international definition.

## Data Availability

Not applicable.
